# CT-Based Assessment of Femoral Stem Anteversion After Intentional Intraoperative Rotational Adjustment of a Single-Wedge Tapered Stem in Total Hip Arthroplasty

**DOI:** 10.3390/jcm15197643

**Published:** 2026-10-02

**Authors:** Soon-Phil Yoon, Byung Woo Cho, Minjae So, Tae Hyeong Kim, Woo-Suk Lee

**Affiliations:** Department of Orthopedic Surgery, Gangnam Severance Hospital, Yonsei University College of Medicine, Seoul 06229, Republic of Korea; yspyonsei0212@gmail.com (S.-P.Y.); chobw0704@yuhs.ac (B.W.C.); majesox@yuhs.ac (M.S.); tae0021@yuhs.ac (T.H.K.)

**Keywords:** total hip arthroplasty, femoral anteversion, single-wedge tapered stem

## Abstract

**Background/Objectives:** Prosthetic anteversion (PA) is generally considered to be constrained by three-point fixation in the sagittal plane. However, previous studies have reported inconsistent changes from native femoral anteversion (NFA) to prosthetic anteversion (PA), ranging from no appreciable change to a mean increase of approximately 8°, suggesting that PA may not be determined by femoral anatomy alone. This study aimed to evaluate whether femoral stem anteversion can be intentionally adjusted towards a target angle and to identify factors associated with failure to achieve the intended correction. **Methods:** We retrospectively reviewed 142 hips in 133 patients who underwent total hip arthroplasty with a target PA of 30°. Hips were divided into increased-anteversion (Group 1) and decreased-anteversion (Group 2) groups according to the intended correction relative to NFA. Correlations between targeted and actual correction angles and factors associated with correction failure were analyzed separately for each group. **Results:** Of 142 hips, 126 were classified as Group 1 and 16 as Group 2. PA significantly increased from NFA in Group 1 (12.72 ± 9.24° vs. 25.17 ± 7.13°; *p* < 0.001) but not in Group 2 (39.13 ± 6.73° vs. 37.84 ± 7.88°; *p* = 0.513). A lower CCR and intended decrease in anteversion were associated with correction failure. In Group 1, targeted and actual correction angles were significantly correlated (r = 0.633, *p* < 0.001). **Conclusions:** Femoral stem anteversion can be intentionally increased using a single-wedge tapered stem, particularly in femora with a high CCR. However, the feasibility of intentionally decreasing anteversion requires further investigation.

## 1. Introduction

Successful total hip arthroplasty (THA) relies on appropriate implant positioning, with the implant version being a critical factor. Excessive antero- or retrotorsion of the components can lead to various complications including impingement, hip instability, dislocation, component wear, and limited range of motion [1]. To address these challenges, Ranawat et al. [2] demonstrated the concept of combined anteversion (CA), defined as the sum of stem anteversion and femoral anteversion. Ranawat et al. recommended a CA values of 25–45° for females and 20–30° for males.

The clinical excellence of this technique has been well-defined in various studies. Since Widmer et al. [3] suggested that the specific range of cup inclination (40–42°), cup anteversion (23–28°), and stem anteversion (calculated using the following formula: 37.3 = Cup anteversion + 0.7 × Femoral anteversion) showed a maximum impingement-free range of motion (ROM), various studies have reported excellent results with similar CA values. For instance, in a study of anteversion measured with a plain radiograph by Jolles et al. [4], patients who had a total anteversion (the sum of cup and stem anteversion) of <40° or >60° had a 6.9 times higher dislocation rate than those who did not. Similarly, a computed tomography (CT)-based study by Nakashima et al. [5] showed that the CA (-) group (CA <40° or >60°) had a 5.8 times higher dislocation rate than the CA (+) group (CA within 40–60°). Another study on THA with the direct anterior approach by Dimitriou et al. [6] also showed a significantly higher dislocation rate when the CA was >60°.

However, previous studies have demonstrated that when using a cementless stem, obtaining ideal prosthetic anteversion (PA) is challenging because of the difficulty in manipulation. This so-called theory of the best-fitting position proposed by D’Lima et al. [7] states that PA is dictated by femoral canal morphology, especially with the cementless stems. Likewise, Dorr et al. [8] specifically suggested the reason for such dictated position: stem version is controlled by the anatomy of the sagittal plane of the femur (neck, anteroposterior isthmus level of the lesser trochanter, and posterior fin of the bone). Bargar et al. [9] agreed on this theory by suggesting a correlation between native femoral anteversion (NFA) and PA. For this reason, Dorr et al. [8] suggested a femur-first CA technique, in which before acetabular cup positioning, the femur broach is first incorporated to confirm the prosthesis version, and subsequently, the acetabular cup is inserted using Widmer’s formula (37.3 = Cup anteversion + 0.7 × Femoral anteversion). However, in cases of excessive femoral anteversion or retroversion, there is a limitation of adjusting anteversion in cup positioning because it may need excessive reaming; additionally, excessive or insufficient cup anteversion can lead to bony protrusion, causing groin pain after THA [10,11]. Although a modular stem may provide greater flexibility in adjusting femoral version, its use is associated with concerns such as corrosion, metallosis, and modular neck fracture [12].

Interestingly, previous studies have reported contrasting findings regarding the relationship between NFA and PA. Worlicek et al. [13] and Klim et al. [1] reported similar mean preoperative and postoperative femoral anteversion. In contrast, Bargar et al. [9] and Imai et al. [14] reported that PA was, on average, 8.7° and 7.7° greater than NFA, respectively. These findings raise the possibility that PA may not be determined solely by native femoral anatomy but may also be influenced by intentional intraoperative rotational adjustment. We therefore hypothesized that PA is not fixed solely by native femoral anatomy but can be intentionally modified through intraoperative rotational adjustment. Accordingly, this study aimed to answer the following questions: (1) When an intentional increase or decrease in stem anteversion relative to NFA is attempted, does PA change in the intended direction? (2) Is there an association between the targeted and actual correction angles? (3) What factors are associated with failure to achieve correction in the intended direction?

## 2. Methods

### 2.1. Ethics Statements

The present study was approved by the Institutional Review Board of our institution (protocol number 3-2023-0160) and was performed in accordance with the ethical standards of the 1964 Declaration of Helsinki.

### 2.2. Study Design and Population

We retrospectively reviewed data that had been prospectively collected. The study included 142 hips from 133 consecutive patients who underwent THA performed by the senior surgeon at our institution between October 2021 and October 2023. All enrolled patients underwent CT examination (GE Revolution CT, GE Medical Systems, Cleveland, OH, USA) within 3 months preoperatively and within 3 days postoperatively at our institution. All patients with proximal femoral fracture were excluded because of potential disruption of the proximal femur anatomy.

### 2.3. Surgical Procedure

**Figure 1:**
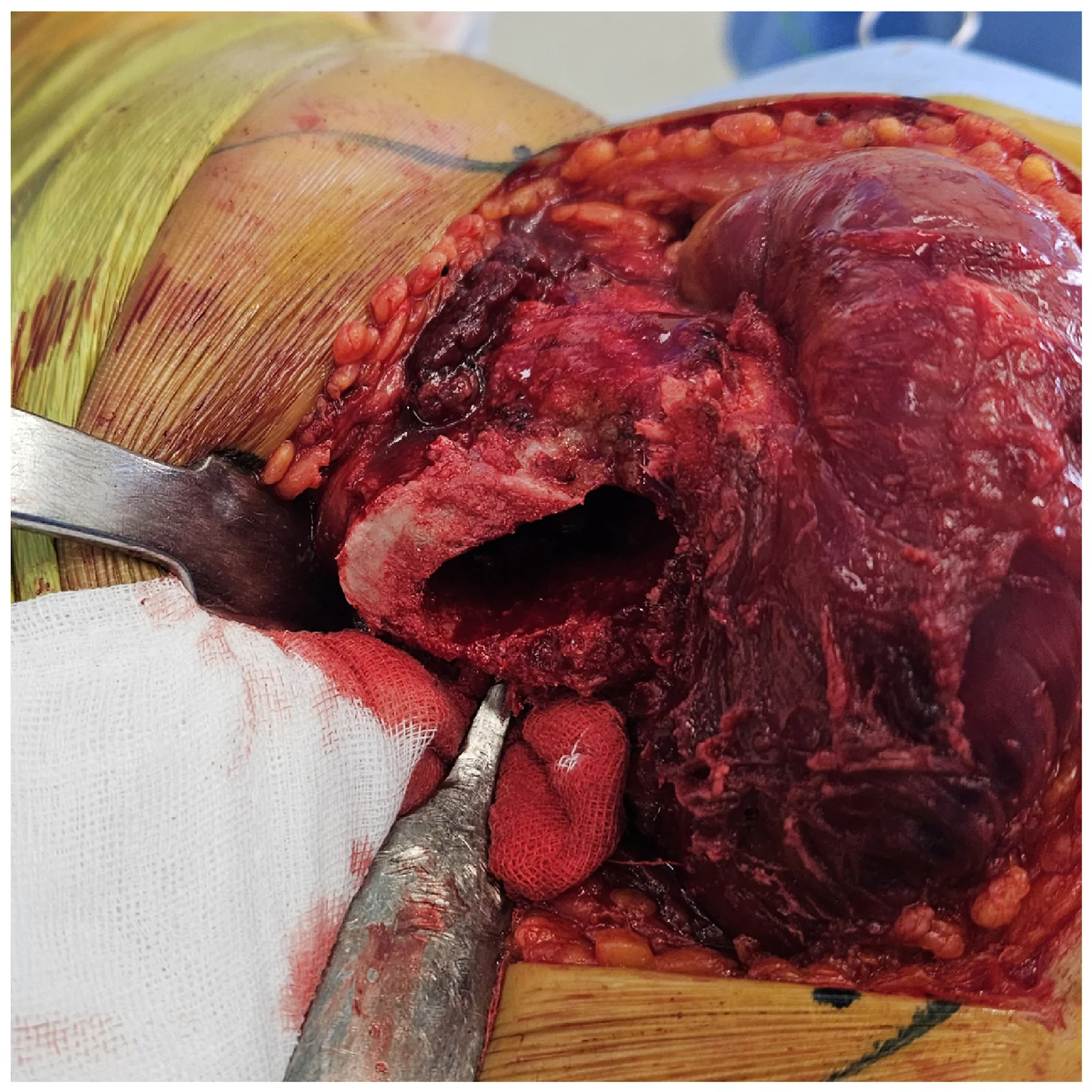
Intraoperative image of final broach. Prosthesis anteversion increased compared with native femoral anteversion.

All surgical procedures used a cementless cup (PINNACLE, DePuy, Warsaw, IN, USA) and a single-wedge tapered stem (Tri-Lock, DePuy, Warsaw, IN, USA). During preoperative planning, we measured the angle between the posterior cortex at the femoral neck cutting level and the posterior condylar line of the femur. The posterior condylar line was measured using the one-plane method in the two-dimensional (2D) measurement technique described by Park et al. [15]. The patients underwent surgery with a posterolateral approach and in a straight lateral position. The femur-first CA technique was performed as suggested by Dorr et al. [8]. Femoral broaching was initiated at approximately 30° of anteversion, based on a previous study demonstrating a favorable impingement-free ROM at this angle (Figure 1) [16]. When further rotational adjustment was limited by the fit of the broach within the proximal femur, additional rotation was not forced, and the stem was implanted in the achievable position. After final broaching, the angle between the final broach and the posterior femoral cortex was measured, and PA was estimated using the preoperatively measured angle between the posterior cortex and posterior condylar line. A previous study showed a correlation between the final broach angle and PA; therefore, the final broach angle was used to estimate PA intraoperatively [9]. Although the final broach angle was measured intraoperatively to estimate PA, the measured value was not separately recorded for subsequent analysis. The acetabular cup was inserted at an angle determined using the CA formula (37.3 = Cup anteversion + 0.7 × femoral anteversion), However, if the acetabular cup could not be placed without bony protrusion of the acetabulum, we inserted the cup with a restricted angle with no bony protrusion. After implant placement, the limb length and ROM including flexion at 90°, adduction at 20°, internal rotation at 40°, extension at 20°, and external rotation at 40° were verified to assess impingement-free ROM and the tendency of dislocation.

### 2.4. Radiologic Evaluation

**Figure 2:**
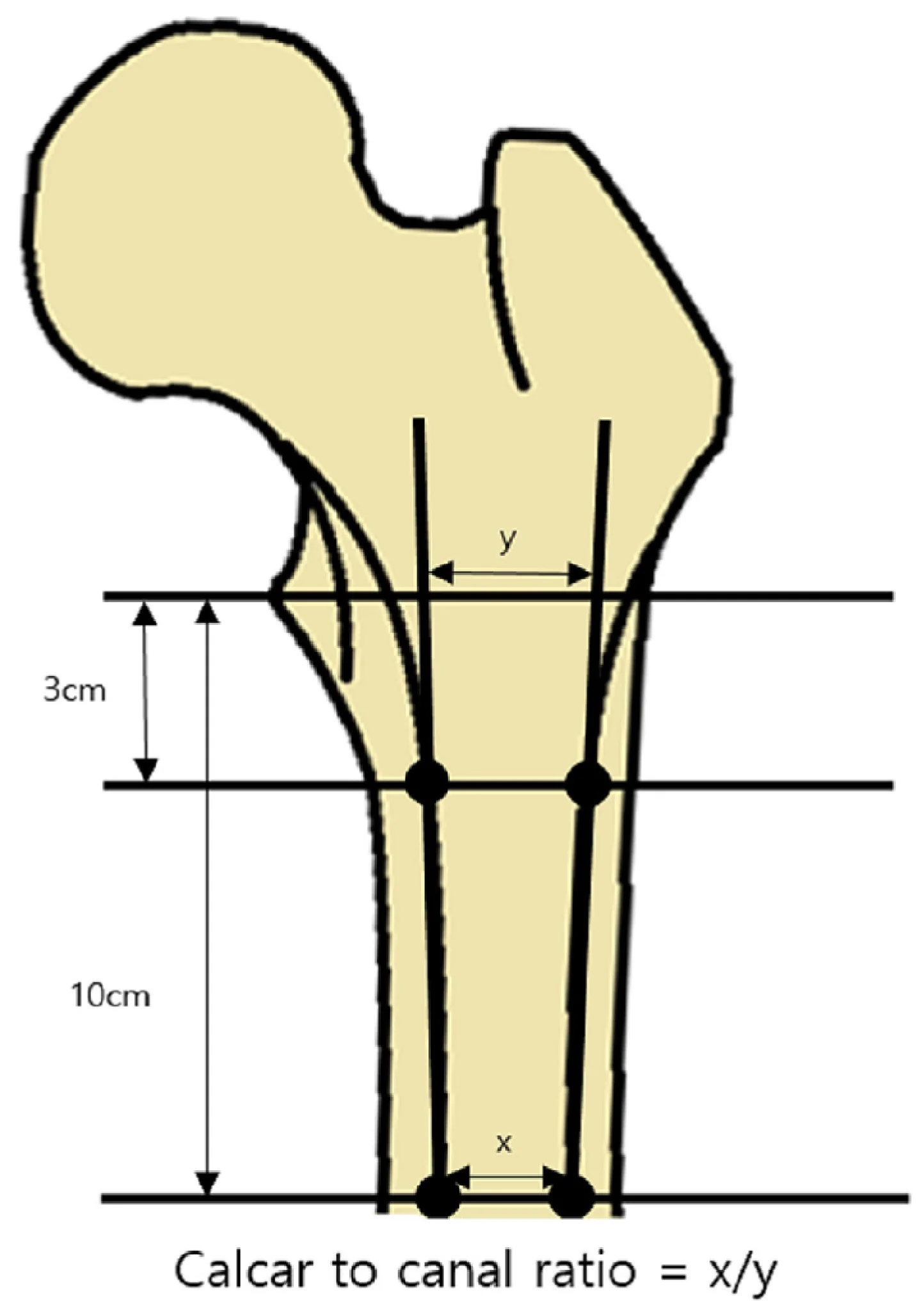
Calcar-to-canal ratio described by Dorr et al. The distal endosteal points are 10 cm from the mid-lesser trochanter line, and the proximal endosteal points are 3 cm from the same line. The calcar-to-canal ratio is 15/30, as shown in the figure.

**Figure 3:**
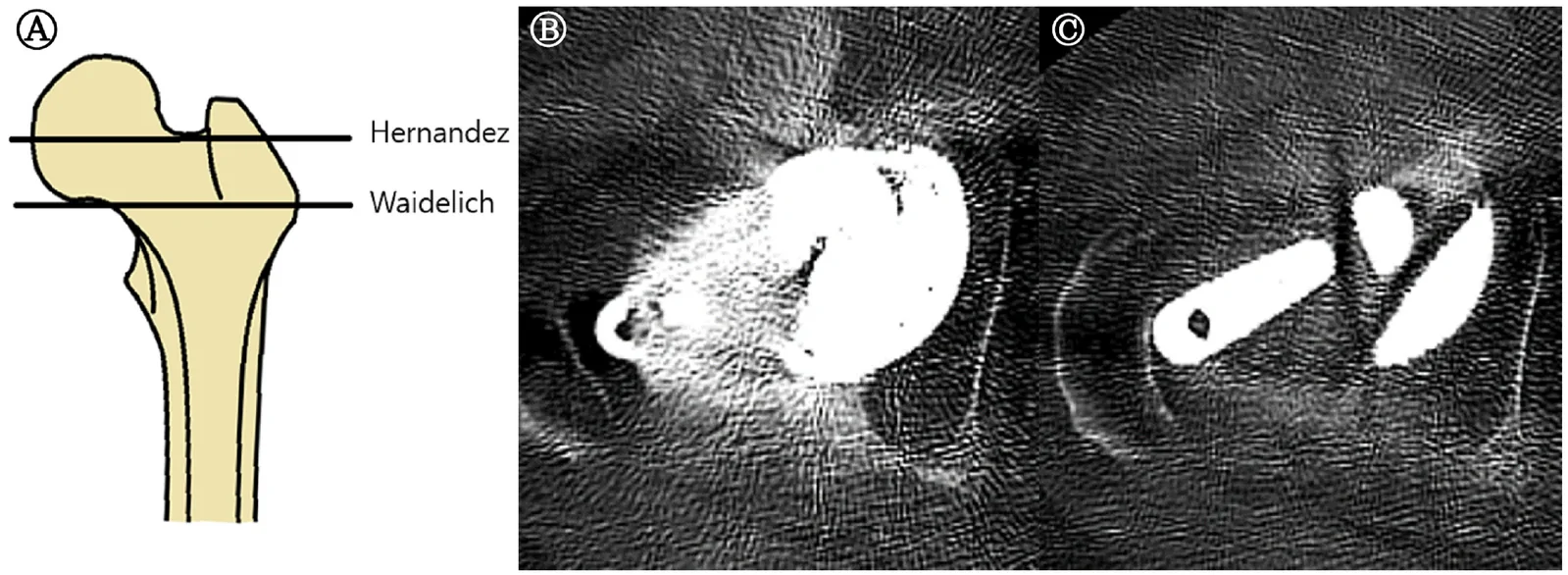
(**A**) Schematic illustration of the axial measurement levels used in the Hernandez and Waidelich methods. Axial computed tomography images obtained at the Hernandez level (**B**) and Waidelich level (**C**).

Preoperative anteroposterior plain radiographs were obtained for each patient to measure the calcar-to-canal ratio (CCR) as an objective parameter reflecting Dorr type (Figure 2) [17]. CT scans were obtained with the patient in the supine position, with the hip joint extended, and the thighs positioned horizontal and parallel to the floor. Axial images of the hip, knee, and ankle were acquired without manipulation with the following parameters: tube voltage, 120 kV; tube current, 100–300 mAs/slice; pitch factor, 0.992; matrix, 512 × 512; and reconstructive thickness, 0.25 mm. Preoperative and postoperative anteversion values were measured using the Waidelich technique to ensure that the same cutting plane (pre- and postoperatively) was obtained (Figure 3) [18]. All radiographic measurements were initially performed by a single observer. To assess measurement reliability, 30 hips were randomly selected and evaluated by a second observer. For this reliability assessment, postoperative PA was measured twice by the second observer, and intraobserver and interobserver reliability were assessed using intraclass correlation coefficients (ICCs).

### 2.5. Data Analysis

Patients were classified into Group 1, in which an increase in PA relative to NFA was intended, and Group 2, in which a decrease in PA relative to NFA was intended. Patient demographics and changes in anteversion after surgery were compared between the groups. A change in PA opposite to the intended direction (a PA lower than NFA in Group 1 or a PA higher than NFA in Group 2) was classified as correction failure for the analysis of factors associated with failure. This definition was intended to assess whether PA changed in the intended direction relative to NFA, rather than whether the predetermined target angle of 30° was achieved. The following factors were evaluated for their association with correction failure: age, sex, body mass index (BMI), diagnosis (categorized as arthritis, subchondral insufficiency fracture [SIF], or osteonecrosis of the femoral head [ONFH]), CCR, and group. The targeted correction angle was calculated using the following formula: 30° − NFA. The actual correction angle was calculated as the difference between PA and NFA. Correlation analysis between the targeted and actual correction angles was performed separately for each group.

### 2.6. Statistical Analysis

All statistical analyses were performed using SPSS (version 25.0; SPSS, Chicago, IL, USA). To assess the potential effect of non-independence among bilateral cases, a sensitivity analysis was performed using one randomly selected hip from each patient with bilateral THA. Continuous variables (including age, BMI, and CCR) were expressed as means with standard deviations (SDs) and compared using independent *t*-tests. Categorical variables (including sex, diagnosis, and correction success) were compared using the chi-squared or Fisher’s exact test. Paired *t*-tests were used to compare NFA and PA. Intraobserver and interobserver reliability were assessed using single-measure intraclass correlation coefficients (ICCs) based on a two-way random-effects model with absolute agreement. Univariable logistic regression analyses were initially performed to identify factors associated with correction failure. Variables showing a significant association in the univariable analyses were subsequently included in the multivariable logistic regression model. A total of 12 correction-failure events were included in the analysis. Finally, correlations between the targeted and actual correction angles were assessed using Pearson’s correlation coefficient because both variables were normally distributed within each group. Correlation coefficients were categorized as poor (0.00–0.20), fair (0.21–0.40), moderate (0.41–0.60), good (0.61–0.80), and excellent (0.81–1.00) [19]. The required sample size was calculated based on the correlation analysis between the targeted and actual correction angles using G*Power software (version 3.1.9.4; Heinrich Heine Universität Düsseldorf, Düsseldorf, Germany). Assuming a correlation coefficient of 0.60, a two-sided α of 0.05, and a power of 80%, approximately 20 hips per group were required.

## 3. Results

The patients’ average age (mean ± SD) was 58.89 ± 14.35 years and average BMI was 23.86 ± 3.25 (kg/m^2^); 95 (66.9%) patients were female. Of the 142 hips, 126 were classified as Group 1, in which an increase in PA relative to NFA was intended, whereas 16 were classified as Group 2, in which a decrease in PA relative to NFA was intended. The correction success rate in Group 1 was 96.8%, which was significantly higher than the 50.0% observed in Group 2 (*p* < 0.001). Additional basic demographic data are presented in Table 1. PA was significantly greater than NFA in Group 1 (*p* < 0.001). The mean NFA angle of Group1 was 12.72° (range −12.48° to 29.9°; SD, 9.24°), whereas that for PA was 25.17° (range 3.55° to 44.3°; SD, 7.13°). In contrast, PA was not significantly different from NFA in Group 2. In Group 2, the mean NFA angle was 39.13° (range 30.8° to 49.7°; SD, 6.73°), whereas that for PA was 37.84° (range 24.85° to 48.3°; SD, 7.88°) (*p* = 0.513) (Table 2).

In the univariable logistic regression analyses, CCR and group were significantly associated with correction failure and were subsequently included in the multivariable logistic regression model. Multivariable logistic regression analysis showed that CCR and group were independently associated with correction failure (Table 3). The odds ratio for an increase in CCR was 0.850 (95% confidence interval [CI], 0.760–0.952; *p* = 0.005), indicating that a higher CCR was associated with a reduced risk of correction failure. The comparison between Groups 1 and 2 showed an odds ratio of 50.012 (95% CI, 8.184–308.492; *p* < 0.001), indicating that Group 2 was associated with substantially higher odds of correction failure than Group 1 (Table 3).

**Figure 4:**
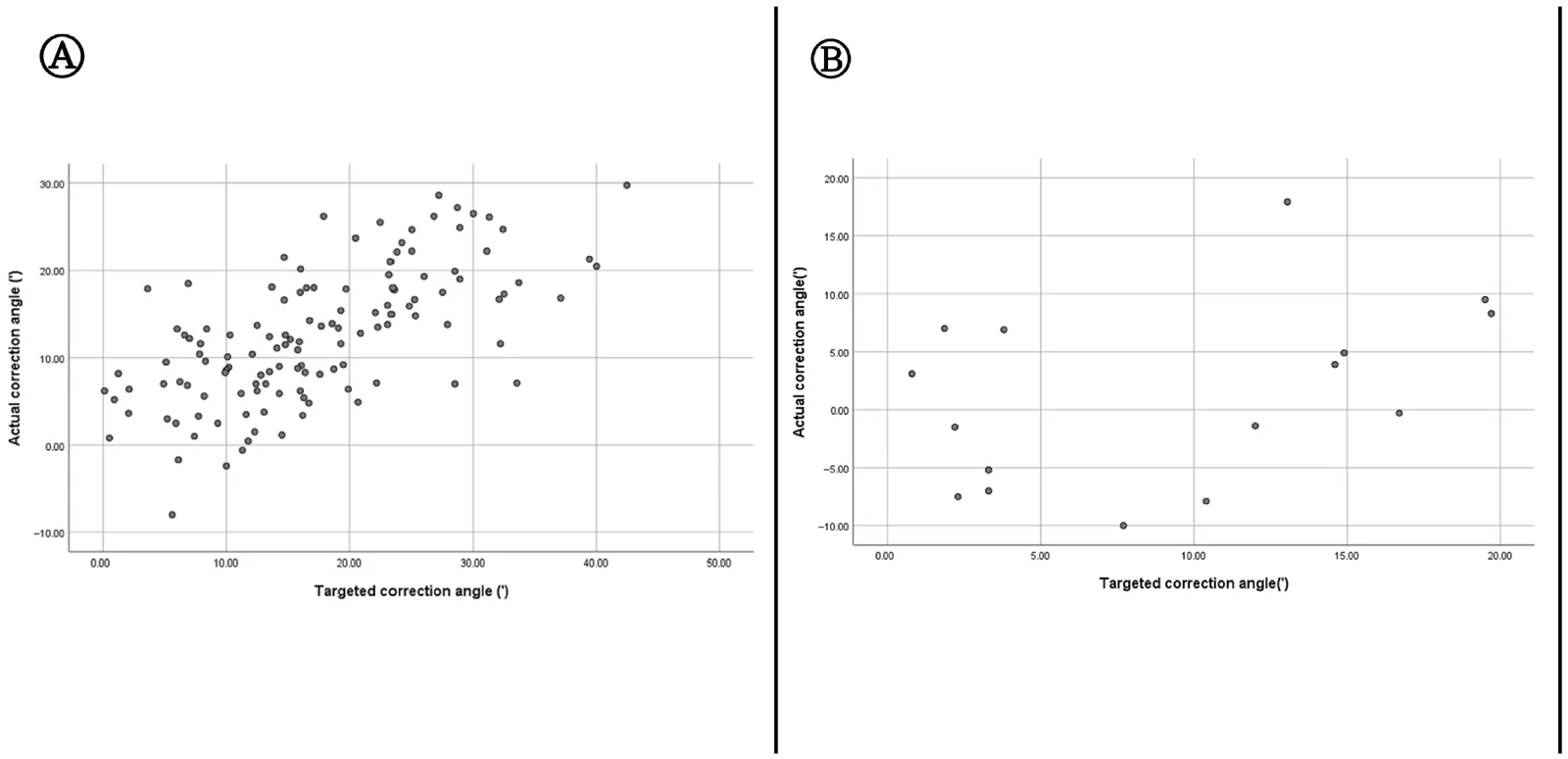
Relationship between targeted correction angle and actual corrected angle in Group 1 (**A**). Correlation coefficient (r) is 0.633 (*p* < 0.001) (**A**). Relationship between targeted correction angle and actual corrected angle in Group 2. Correlation coefficient (r) is 0.413 (*p* = 0.111) (**B**).

The targeted and actual correction angles showed a good correlation in Group 1 (r = 0.633, *p* < 0.001; Figure 4A), indicating a significant association between the targeted and actual correction angles. However, no significant correlation was observed in Group 2 (r = 0.413, *p* = 0.111; Figure 4B). Given the limited sample size of Group 2, this finding should be interpreted cautiously.

The intraobserver and interobserver ICCs for postoperative PA measurements were 0.945 and 0.935, respectively. A sensitivity analysis including one randomly selected hip per patient yielded results consistent with those of the primary analysis.

## 4. Discussion

The most important finding of our study is that PA may be intentionally modified relative to the best-fitting position when an increase in anteversion is intended with a single-wedge tapered stem. A significant increase in PA relative to NFA was observed in Group 1, whereas no significant difference between NFA and PA was observed in Group 2. Because no established threshold exists for defining successful rotational correction of femoral stem anteversion, correction success was operationally defined according to the direction of change relative to NFA. Therefore, “correction success” in the present study indicates that PA changed in the intended direction and should not be interpreted as successful achievement of the predetermined target angle of 30°. Furthermore, we found a correlation between the targeted and actual correction angles in Group 1. This flexibility may be beneficial when an increase in PA is required in patients with low NFA. Additionally, our study revealed that a low CCR was associated with higher odds of correction failure. Thus, rotational adjustment appeared to be more constrained in Dorr type A femurs and more flexible in Dorr type C femurs. This finding may provide a possible explanation for the excessive femoral anteversion reported by Watanabe et al. [20], indicating that the best-fitting position is more confirmative in Dorr type A femurs with an angle that differs from the NFA.

The best-fitting position theory, which describes constraints on femoral rotation resulting from three-point fixation in the sagittal plane of the femur, has been widely accepted in previous studies [7,8,9]. However, previous studies have reported conflicting findings regarding the relationship between PA and NFA (Table 4). Worlicek et al. [13] and Klim et al. [1] reported similar mean NFA and PA. In contrast, Bargar et al. [9] and Imai et al. [14] reported mean PA values that were 8.7° and 7.7° greater than NFA, respectively. Interestingly, the intended stem orientation also differed among previous studies. Klim et al. [1] aimed for a femoral anteversion of 15°, yet the mean postoperative PA remained similar to the mean NFA. In contrast, Imai et al. [14] reported a mean planned stem version greater than the mean NFA, and the postoperative PA was also greater than the NFA. These findings, together with our results, suggest that postoperative PA may be influenced not only by native femoral anatomy but also by intentional rotational adjustment during stem insertion.

A possible explanation for the differences in rotational adjustment between Groups 1 and 2 and the higher odds of correction failure in patients with low CCR may be related to proximal femoral canal morphology. Khanuja et al. [21] demonstrated that a single-wedge stem may engage only the distal part of a narrow femoral diaphysis, where no porous coating is present. Moreover, they demonstrated that osseointegration may not occur in the proximal porous-coated metaphyseal area. A previous long-term follow-up study of a single-wedge stem showed that even with a metaphyseal-fixation stem, stress shielding occurred in the proximal femur [22]. Therefore, the association between a low CCR and correction failure observed in our study may be related to the location and extent of stem–bone engagement within the femoral canal. Additionally, our multivariable logistic regression analysis showed that a lower CCR was independently associated with higher odds of correction failure. Further long-term follow-up is needed to determine whether postoperative radiographic changes, such as stress shielding and distal cortical thickening, are associated with the pattern of stem–bone engagement and correction failure.

Our findings may provide a basis for further investigation of the cup-first CA technique introduced by Masumoto et al. [23]. They inserted the acetabular cup first, and using a navigation system, they measured cup orientation and used this information to determine stem anteversion according to CA formula. Compared with the traditional femur-first CA technique, this technique is advantageous in avoiding excessive reaming and protrusion of the cup; however, in the case of a cementless stem, the inability to manipulate PA is considered an obstacle. Our findings suggest that intentional adjustment of PA with a cementless stem may provide an opportunity to further investigate the applicability of this technique. Several limitations should be considered when attempting intentional adjustment of PA. First, decreasing PA relative to NFA may be more difficult than increasing it. Second, in Dorr type A femurs, rotational adjustment may be more constrained by the best-fitting position. Third, although a significant correlation was observed between the targeted and actual correction angles in Group 1 (r = 0.633), the relationship was not sufficiently strong to indicate reliable achievement of the predetermined target angle. Moreover, although multiple cases targeted a correction angle of more than 30°, not a single case succeeded in manipulating PA by more than 30°. Thus, in excessively anteverted or retroverted femora, this technique should be used cautiously.

Our study has some limitations. First, the sample size of Group 2 (*n* = 16) was smaller than the required sample size estimated for the correlation analysis, which may have limited the ability to detect a significant association in this group. Therefore, the nonsignificant findings in Group 2 should be interpreted cautiously. However, hips requiring a decrease in anteversion were relatively uncommon because the average NFA in the Korean population is markedly lower than 30° [24]. Consequently, the number of patients eligible for Group 2 was inherently limited. Second, we used 2D axial CT to measure anteversion, which may be less accurate than using a 3D model [19]. Third, all procedures were performed by a single experienced surgeon at a single institution using the same implant system and posterolateral approach. Although this provided procedural consistency, the findings may not be directly generalizable to other surgeons, stem designs, surgical approaches or patient populations. Therefore, future large-scale studies, particularly those including a larger number of hips with high NFA and using 3D modeling analysis, are required to confirm our findings. Fourth, 9 patients contributed both hips, and although a sensitivity analysis using one randomly selected hip per patient yielded consistent results, residual non-independence cannot be entirely excluded.

In conclusion, intentional rotational adjustment of a single-wedge tapered stem may allow modification of PA in the intended direction, particularly when an increase in anteversion is required and in femora with a high CCR. However, the feasibility of decreasing anteversion requires further investigation because of the limited number of cases, and these findings should not be interpreted as demonstrating the reliable achievement of a predetermined target angle.

## Figures and Tables

**Table 1:** Patient demographics and comparison of Groups 1 and 2.

	Total (*N* = 142)	Group1 (*N* = 126)	Group2 (*N* = 16)	*p*-Value
Age (years)	58.89 ± 14.35	59.39 ± 14.42	55 ± 13.52	0.25
Females (%)	95 (66.9)	83 (65.9)	12 (75)	0.465
BMI (kg/m^2^)	23.86 ± 3.25	23.83 ± 3.17	24.14 ± 3.98	0.723
Diagnosis (%)				0.457
Arthritis	77 (54.2)	68 (54)	9 (56.3)	
SIF	11 (7.7)	11 (8.7)	0	
ONFH	54 (38)	47 (37.3)	7 (43.8)	
Calcar to canal ratio %	59.65 ± 8.69	59.9 ± 8.72	57.69 ± 8.4	0.338
Correction succession (Success, %)	130 (91.5)	122 (96.8)	8 (50)	<0.001

All quantitative variables are presented as mean ± standard deviation. BMI, body mass index; SIF, subchondral insufficiency fracture; ONFH, osteonecrosis of the femoral head.

**Table 2:** Comparison of pre- and postoperative anteversion change in Groups 1 and 2.

	Preoperative Anteversion	Postoperative Anteversion	*p*-Value
Group 1 (°)	12.72 ± 9.24	25.17 ± 7.13	<0.001
Group 2 (°)	39.13 ± 6.73	37.84 ± 7.88	0.513

All quantitative variables are presented as means ± standard deviations.

**Table 3:** Multiple logistic regression analysis of risk factors for correction failure.

Variables	Univariable		Multivariable	
	OR (95% CI)	*p*-Value	OR (95% CI)	*p*-Value
Age (years)	1.004 (0.963–1.047)	0.845		
Sex	0.378 (0.079–1.799)	0.221		
BMI (kg/m^2^)	0.937 (0.772–1.138)	0.512		
Diagnosis (%)				
Arthritis	Ref			
SIF	1.201 (0.349–4.178)	0.766		
ONFH	1.138 (0.129–10.875)	0.882		
Calcar to canal ratio %	0.885 (0.814–0.962)	0.004	0.850 (0.760–0.952)	0.005
Group (%)				
1	Ref		Ref	
2	30.5 (7.542–123.34)	<0.001	50.012 (8.184–308.492)	<0.001

All quantitative variables are presented as means ± standard deviations. OR, odds ratio; CI, confidence interval; BMI, body mass index; SIF, subchondral insufficiency fracture; ONFH, osteonecrosis of femoral head.

**Table 4:** Comparison of previous studies evaluating native femoral anteversion and postoperative prosthetic anteversion.

First Author	No. of Hips	Stem	Stem Design	Assessment Method	NFA → PA (°)	NFA–PA Correlation
Klim et al. [1]	89 *	ANA.NOVA Alpha Schaft Proxy (ImplanTec, GmbH, Moedling, Austria)	Short stem	Pre-/postoperative CT-based 3D measurement	22.4 ± 11.0 → 23.4 ± 10.1	Not reported
Worlicek et al. [13]	55	Corail (DePuy, Warsaw, IN, USA)	Single-wedge	Imageless navigation	14.9 ± 9.3 → 13.0 ± 9.5 (−1.9)	r = 0.39, *p* < 0.003
Bargar et al. [9]	46	Fiber Metal Taper (Zimmer, Warsaw, IN, USA)	Double-wedge	CT-based 3D templating (ORTHODOC)	13.8 ± 7.9 → 22.5 ± 8.5 (+8.7)	r = 0.831, *p* < 0.0001
Imai et al. [14]	65	PerFix 910 HA (Kyocera, Medical, Osaka, Japan)	Double-wedge	CT-based 3D planning and postoperative CT	24.3 ± 12.2 → 32.0 ± 10.3 (+7.7)	r = 0.63, *p* < 0.01

NFA, native femoral anteversion; PA, prosthetic anteversion. * Only the short-stem group, for which both preoperative and postoperative femoral anteversion measurements were available, is presented here.

## Data Availability

The data presented in this study are available on request from the corresponding author due to privacy and ethical restrictions.
